# Modulation of Hepatitis C Virus RNA Accumulation and Translation by DDX6 and miR-122 Are Mediated by Separate Mechanisms

**DOI:** 10.1371/journal.pone.0067437

**Published:** 2013-06-24

**Authors:** Adam Huys, Patricia A. Thibault, Joyce A. Wilson

**Affiliations:** 1 Department of Microbiology and Immunology, University of Saskatchewan, Saskatoon, Canada; 2 Vaccine and Infectious Disease Organization-International Vaccine Centre (VIDO-InterVac), University of Saskatchewan, Saskatoon, Canada; University of Cincinnati College of Medicine, United States of America

## Abstract

DDX6 and other P-body proteins are required for efficient replication of Hepatitis C Virus (HCV) by unknown mechanisms. DDX6 has been implicated in miRNA induced gene silencing, and since efficient HCV replication and translation relies on the cellular microRNA, miR-122, we hypothesized that DDX6 had a role in the mechanism of action of miR-122. However, by using multiple HCV translation and replication assays we have found this is not the case. DDX6 silencing decreased HCV replication and translation, but did not affect the ability of miR-122 to stimulate HCV translation or promote HCV RNA accumulation. In addition, the negative effect of DDX6 silencing on HCV replication and translation was not dependent on miR-122 association with the HCV genome. Thus, DDX6 does not have a role in the activity of miR-122, and it appears that DDX6 and miR-122 modulate HCV through distinct pathways. This effect was seen in both Huh7.5 cells and in Hep3B cells, indicating that the effects are not cell type specific. Since infections by other viruses in the *Flaviviridae* family, including Dengue and West Nile Virus, also disrupt P-bodies and are regulated by DDX6, we speculate that DDX6 may have a common function that support the replication of several Flaviviruses.

## Introduction

Processing bodies (P-bodies) are transient cellular compartments where mRNAs are degraded and sometimes stored [1], [2], [3]. P-bodies are composed of an array of proteins such as mRNA de-capping, de-adenylating, and RNA exoribonuclease enzymes, many of which have been implicated in miRNA suppression and mRNA turn-over. The composition, location, and number of P-bodies in a cell is dynamic and based on mRNA degradation requirements [2]. Fewer P-bodies are found in cells under conditions of increased mRNA translation, due to a reduced need for mRNA degradation, and conversely, when mRNA degradation is promoted by impeded cellular translation, P-bodies are found in greater numbers and increased size.

microRNA-mediated mRNA silencing involves both the suppression of translation, and induction of mRNA degradation [4]. The process of miRNA silencing involves binding of miRNA and associating proteins Argonaute (Ago) and GW182. GW182 then associates with Poly(A)-binding protein (PABP) and several host protein components of the deadenylation complexes, which is believed to be at least part of the mechanism by which miRNAs suppress translation and promote mRNA degradation [5], [6]. P-bodies are the likely sites of miRNA-induced mRNA degradation since they contain high concentrations of miRNAs, Ago and GW182 [7]. The resident P-body protein DDX6 (RCK, p54) is essential for the assembly and maintenance of P-bodies, and depletion of DDX6 inhibits P-body formation, even when stimulated by arsenite, a robust translation inhibitor and P-body inducer [8]. DDX6 and its homologues from different species such as *Saccharomyces cerevisiae* (Dhh1), *Xenopus laevis* (Xp54) and *Caenorhabditis elegans* (CGH-1) are members of the DEAD-box RNA helicase family and bind to RNA with high affinity. Once bound, DDX6 has the ability to modify the secondary structure of the RNA in an energy independent and dependent manner [9], [10], [11]. In addition, the *S. cerevisiae* DDX6 homologue, Dhh1, stimulates decapping of mRNAs by decreasing the rate of translation, presumably by exposing the cap to decapping enzymes [12], [13]. DDX6 is also believed to enhance miRNA gene suppression, as its knockdown leads to an alleviation of miRNA suppression in HeLa cells [14].

Some RNA viruses have been shown to disrupt P-bodies during infection, while others appear to use them as sites for replication, assembly, and release; thus, the relationship between P-bodies and RNA viruses has been the focus of extensive study [15], [16], [17], [18], [19]. Of particular interest is the Hepatitis C Virus (HCV) a human pathogen that causes liver cirrhosis, liver failure and hepatocellular carcinoma. HCV, a 9.6 kb positive strand RNA virus and member of the *Flaviviradae* family, has been demonstrated to alter P-body distribution during infections [20]. The role of the redistribution of P-bodies during HCV infection is unknown, but P-bodies themselves do not appear to be required for HCV replication [20], [21]. However, knocking down P-body proteins Lsm-1, PatL-1, Ge-1, GW182, Ago2, and DDX6 results in reduced HCV replication, indicating a direct or indirect role for these proteins in supporting the HCV life cycle [22], [23], [24], [25]. DDX6 protein levels are also frequently elevated in HCV-associated carcinomas, while being down-regulated in other liver carcinomas, suggesting a possible role in HCV-induced liver pathology [26]. Knocking down DDX6 in cell culture reduces HCV RNA replication, but there is conflicting evidence regarding whether DDX6 silencing decreases HCV translation [22], [24]. Scheller *et al.* [24] found that DDX6 knockdown reduced HCV translation levels while Jangra *et al.* [22] observed no effect on translation. DDX6 has been demonstrated to co-precipitate with HCV core protein, and through binding to core, associate with HCV RNA [22]. DDX6 also localizes near HCV replication centers, suggesting it may play a role in trafficking or regulating HCV RNA [20], [22], [23]. Thus the function of DDX6 in HCV replication needs further study in order to better understand the relationship and its possible link to hepatocellular carcinoma.

HCV requires miR-122, an abundantly expressed liver-specific miRNA, to efficiently establish an infection [27], however the mechanism of action of miR-122 is unknown. The relationship between miR-122 and HCV is unusual in that unlike conventional miRNA-mRNA interactions, which normally take place between the miRNA seed region (the 5′ nucleotides 2–8) and sequences in the 3′ UTR of mRNA, miR-122 binds to two tandem seed binding sequences within the HCV 5′ UTR [27], [28]. In addition, instead of down-regulating translation and RNA stability, miR-122 promotes viral RNA accumulation, mostly by stabilizing the HCV genome, although it can also stimulate translation [29], [30], [31], [32]; and a direct role for miR-122 in promoting viral genome replication has not been ruled out [33]. Like in miRNA suppression, annealing between the seed sequences of miR-122 to the HCV genome is required for activity, but unusually, so too are some of the nucleotides outside of the seed sequence; in particular, nucleotides 15 and 16 at the miR-122 3′ end anneal to sequences at the 5′ end of the HCV genome, creating an RNA overhang which likely protects the uncapped HCV 5′ terminus from access by RNA degradation enzymes [34]. Lastly, the space between the two miR-122 binding sites, and Ago2 are also crucial for miR-122 augmentation of HCV RNA accumulation [25], [28], [35]. Importantly, using miR-122 antagonists to block the activity of miR-122 in both chimpanzees and humans dramatically decreased serum HCV titres, making miR-122 a promising target for antiviral treatment and highlights the importance of miR-122 and the miRNA pathway in HCV life cycle [36]. As a result, efforts to understand the mechanism of action of miR-122 are ongoing.

Because DDX6 knockdown attenuates miRNA suppression activity, we hypothesized that DDX6 may modulate HCV replication and translation by mediating the activity of miR-122, or *vice versa*. Jangra *et al.* showed that DDX6 knockdown did not affect the ability of miR-122 to augment HCV replication [22], and our goal was to expand on these studies by using several model HCV replication and translation assay systems to confirm whether there is, or is not, a connection between the influence of DDX6 and miR-122 on the HCV life cycle. First, our observations confirm that DDX6 knockdown modulates both HCV translation and replication. Next we show that the DDX6 is not required for miR-122 to affect translation, nor is miR-122 annealing required for DDX6 to affect HCV translation. In addition, by using various assays to analyze HCV RNA accumulation, including a novel assay in which HCV replicates independently from miR-122 [37], we have confirmed both that DDX6 is not required for the activity of miR-122 on HCV RNA accumulation, and that miR-122 is not required for the influence of DDX6 on HCV replication. These data are strong indicators that, although both DDX6 and miRNAs are located within P-bodies and are implicated in miRNA suppression activity, they do not affect HCV replication and translation through a common mechanistic pathway.

## Materials and Methods

### Cell Culture

Huh7.5 cells [38] were used for all experiments unless otherwise stated, and were grown in D-MEM supplemented with 10% fetal bovine serum, 0.1 nM non-essential amino acids (Wisent, Montreal, Canada), and 100 units/ml Pen/Strep (Life Technologies, Burlington ON, Canada). Hep3B cells (ATCC number HB-8064) are a human hepatoma cell line and were grown under the same conditions as Huh7.5 cells.

### Plasmids and DNA probes

The pSGR JFH-1 Fluc WT sub-genomic replicon was provided by Dr. T. Wakita [39] and the full-length genome constructs pJ6/JFH-1 (p7-Rluc2A), pJ6/JFH-1 (p7-Rluc2A) GNN, (herein called J6/JFH-1 Rluc and J6/JFH-1 Rluc GNN) were provided by Dr. Charles M. Rice [40]. pJ6/JFH-1 Rluc p34, pJ6/JFH-1 Rluc p34 GNN, and pSGR JFH-1 p3 were described previously [25], [37]. The plasmids pT7Luc and pRL-TK were obtained from Promega Co. (Madison, WI). pLuc-122×2 and pLuc-122×2 S1+S2:p3–4 were kindly provided by Dr. Peter Sarnow [28], and the plasmid pRL-TK CXCR4 4× was provided by Dr. Tariq M. Rana [14].

### Small interfering RNAs (siRNA), duplex microRNA (miRNA), and miR-122 antagonist sequences

All small RNAs were synthesized by Thermo-scientific Dharmacon Inc (Lafayette, CO). The target sequence for the siRNA used for siControl was GAGAGUCAGUCAGCUAAUCA and siDDX6 was ACCCGAGGUAUUGAUAUACAA. The sequence for the duplex miRNA were as follows: miControl, GAGAGUCAGUCAGCUAAUCA; miCXCR4 antisense, UGUUAGCUGGAGUGAAAACUU; miCXCR4 sense, GUUUUCACUCCAGCUAACACA; miR-122, UGGAGUGUGACAAUGGUGUUUGU; miR-122p3 UGCAGUGUGACAAUGGUGUUUGU; miR-122p3–4 UGCUGUGUGACAAUGGUGUUUGU; miR-122*, AAACGCCAUUAUCACACUAAAUA. miR-122, miR-122p3, and miR-122p34 duplex were formed by annealing the indicated miRNA guide strand with miR-122*. The miR-122 antagonist, hsa-miR-122a miRIDIAN microRNA Hairpin Inhibitor, bears a proprietary sequence.

### *In vitro* RNA transcription

HCV RNA transcripts were prepared from XbaI-linearized plasmids as described previously [25] by using the MEGAScript T7 High Yield Transcription Kit, while pRL-TK was linearized with BglII to generate Rluc mRNA and pT7 luciferase was linearized with XmnI to generate Fluc mRNA using mMessage mMachine T7 Transcription Kit (Life Technologies, Burlington, ON, Canada) [37].

### Electroporation of Huh 7.5 and Hep3B cells

Huh7.5 and Hep3B cells were electroporated as previously described [37].

### Transient HCV replication assays

Huh7.5 cells were initially electroporated with 60 pmol of siRNA to achieve knockdown of the desired protein. Three days post-electroporation, cells were electroporated again with 1 µg of HCV RNA, 1 ug of control mRNA, 60 pmol of siRNA, and 60 pmol of miRNA, if applicable. Electroporated cells were re-suspended in 8 mL of media and plated for luciferase assays, protein analysis, RNA analysis, and cell number assays. Cells where harvested 3 days post-electropoartion unless otherwise specified. An additional luciferase assay sample was harvested at 1 hour post-electroporation to confirm electroporation efficiency. Experiments with Hep3B cells were conducted using the same method, except that 5 µg of HCV RNA was used instead of 1 µg.

### Transient HCV translation assays

Huh7.5 cells were electroporated on day 0 with 60 pmol of siRNA in order to silence the gene of interest. The cells were electroporated again on day 3 with 5 ug of J6/JFH-1 RLuc GNN or J6/JFH-1 RLuc p34 GNN RNA and 1 ug of Fluc capped mRNA. Immediately prior to the second electroporation a sample of the cells was harvested for analysis of protein knockdown by western blot. After the second electroporation, cells were plated for luciferase assays, RNA analysis and cell number assays and were harvested 3.5 hours post-second-electroporation.

### Translation suppression assays

Huh7.5 cells were electroporated with 60 pmol of siRNA on day 0, resuspended in 8 ml of media and then 300 µl was seeded into each well of a 24-well plate. Two days post-electroporation the cells were transfected with 100 ng of plasmids pLuc-122×2 or pLuc-122×2 S1+S2:p3–4, and pRL-TK; or pRL-TK CXCR4 4× and pLuc-122×2 S1+S2:p3–4 [28] and 0.05–5 pmol of miRNA per well using Lipofectamine 2000 (Life Technologies, Burlington, ON, Canada) and the suggested protocol. Cells were harvested 24 hours post-transfection for luciferase assays.

### Luciferase assays

Luciferase assays were performed as previously described [25]. Briefly, the cells where washed with phosphate-buffered saline (PBS), and lysed into 100 µl of the appropriate lysis buffer. Luciferase levels were assayed by using Renilla Luciferase, Firefly Luciferase, or Dual Luciferase assay kits (Promega Co., Madison. WI, USA) and light emission was measured by the Glomax 20/20 Luminometer (Promega Co., Madison, WI, USA).

### Cell number assay

Cell numbers were calculated three days post-electroporation or 24 hours post-transfection using WST-1 reagent. The WST-1 assay was performed according to the protocol provided by Roche (Roche Canada, Mississauga, ON, Canada). Cell numbers were determined by comparing them to a standard curve.

### RNA purification

Cells were harvested into 1 mL of Trizol (Life Technologies, Burlington, ON, Canada) and RNA purified using the manufacturer's recommended protocol.

### Northern blot analysis

Northern blots were conducted as previously described [25].

### Real-time PCR analysis of RNA

RNA was reverse-transcribed using the iScript reverse transcription kit (Bio-Rad Inc., Missassauga, ON, Canada). DDX6 mRNA (Life Technologies, Hs00898913) and GAPDH mRNA (used as internal control [#4352934E]) levels were quantified using TaqMan (Life Technologies) probes, primers, and protocol. HCV and Fluc RNA levels where determined using primers directed towards the Renilla (RLuc) gene in the reporter HCV genomic RNA, and Fluc in the control mRNA as described previously [41].

### SDS-Page and western blot analysis

Protein samples were collected by lysing equal number of cells in SDS-PAGE protein lysis buffer (10% SDS, 5% beta-mercaptoethanol, 20% glycerol, 0.2 M Tris-HCl, pH 6.8, 0.05% bromophenol blue). Samples were electrophoresed through 10% SDS-polyacrylamide gels and transferred onto a Hybond-C Extra nitrocellulose membrane (GE Healthcare, Mississauga, ON, Canada). Blots were probed with primary antibodies (1∶5000) mouse monoclonal anti-actin (AC-15; Abcam, Cambridge, MA, USA) and (1∶5000) rabbit anti-RCK (DDX6 antibody; Bethyl labs, Montgomery, TX, USA). Blots were then probed with (1∶1000) anti-mouse (800 nm) and anti-rabbit (680 nm) infrared dye-linked secondary antibody (Li-Cor Biosciences, Lincoln, NE) and visualized using a Li-Cor Odyssey infrared imager and knockdown was determined using Odyssey Infrared Imaging System Application Software Version 3.0.

### Fluorescence microscopy

Huh7.5 cells were plated onto an 8-chamber slide. The cells were fixed using paraformaldehyde and permeabilized with 0.5% Triton X-100. The cells were then exposed to primary antibody, human IC6 polyclonal antibody [42] (a gift from Marvin Fritzler), followed by secondary antibody, Alexa fluor®594 Goat anti-human IgG (H+L) (Life Technologies, Burlington, ON, Canada). Fluorescence images were obtained using a Zeiss axiovert 200 M inverted microscrope at a magnification of 63×10 and the Axiovision 4.6 software.

### Statistical analyses

Data are presented as the average of least three independent experiments, unless otherwise indicated, and error bars represent the standard deviation (SD). Data analysis was carried out with Prism 5 software. P values, unless otherwise indicated were calculated by using Student *t*-test *P<0.05, **p<0.01, ***p<0.001, ****p<0.0001.

## Results

### Depletion of DDX6 reduces P-body abundance

**Figure 1:**
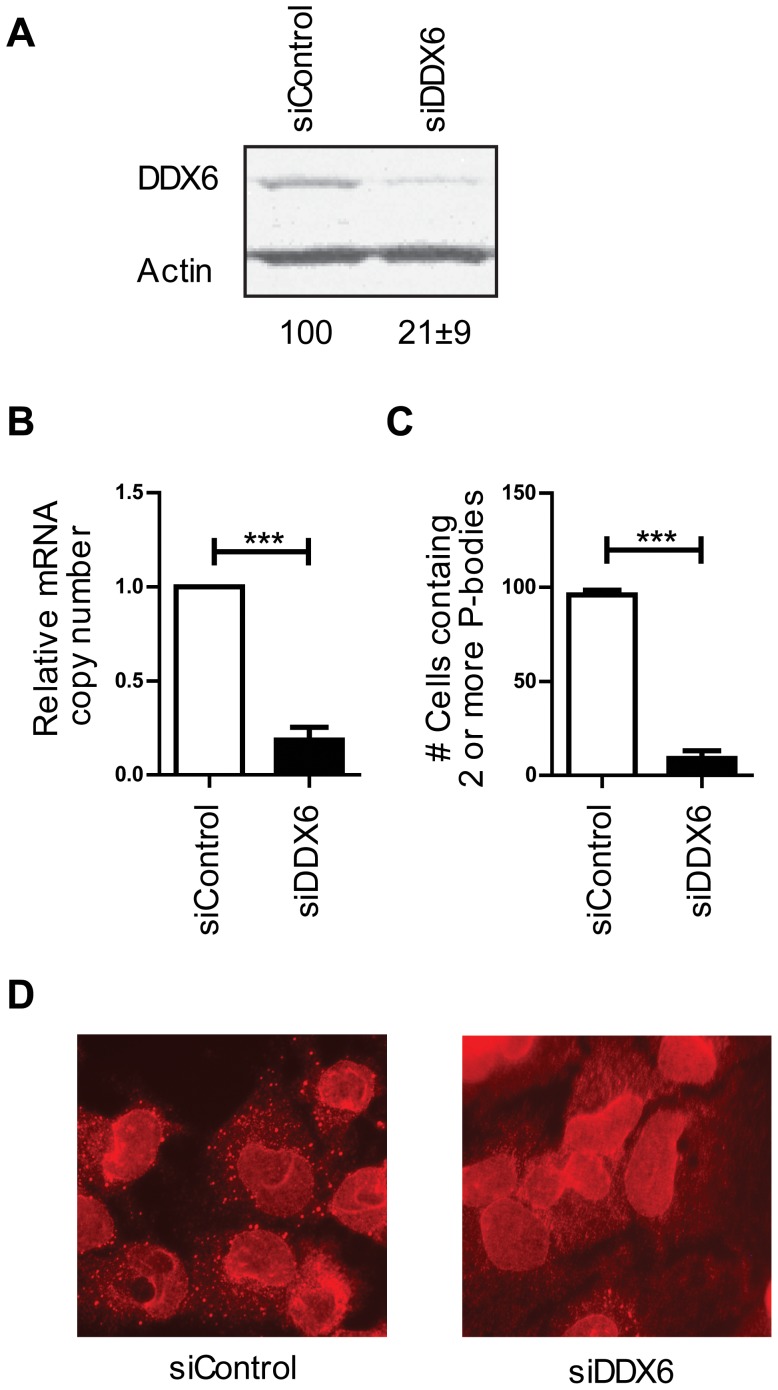
DDX6 specific siRNA, siDDX6, depletes cells of DDX6 and disrupts P-body formation. (A) Western blot analysis shows siDDX6 protein levels in siControl and siDDX6 treated cells. The values represent the average relative DDX6 protein levels and standard deviation from western blot analyses of 12 independent experiments (B) qRT-PCR analysis show that siDDX6 depletes cells of DDX6 mRNA (C) Enumeration of cells containing two or fewer P-bodies after siRNA treatment. (D) Immunoflorescence staining of P-bodies by staining for the P-body protein (Ge-1) in DDX6 depleted cells and control cells.

Following DDX6-specific or control siRNA treatment, levels of DDX6 protein, mRNA and P-bodies were evaluated by western blot, qRT-PCR, and microscopic analysis. Huh7.5 cells treated with DDX6 siRNA expressed 79%±9% less DDX6 protein and 81% less mRNA, compared to cells treated with control siRNA (Fig.1A and B). DDX6 knockdown also significantly reduced the abundance of visible P-bodies (Fig. 1D). P-body disruption was enumerated by assessing the numbers of P-bodies per cell in random fields of 100 cells. Only 9% of the Huh7.5 cells treated with siDDX6 contained two or more P-bodies in contrast to 96% in cells that had been treated with control siRNA (Fig. 1C).

### Silencing of DDX6 attenuates replication of both full-length and sub-genomic HCV replicon RNA

**Figure 2:**
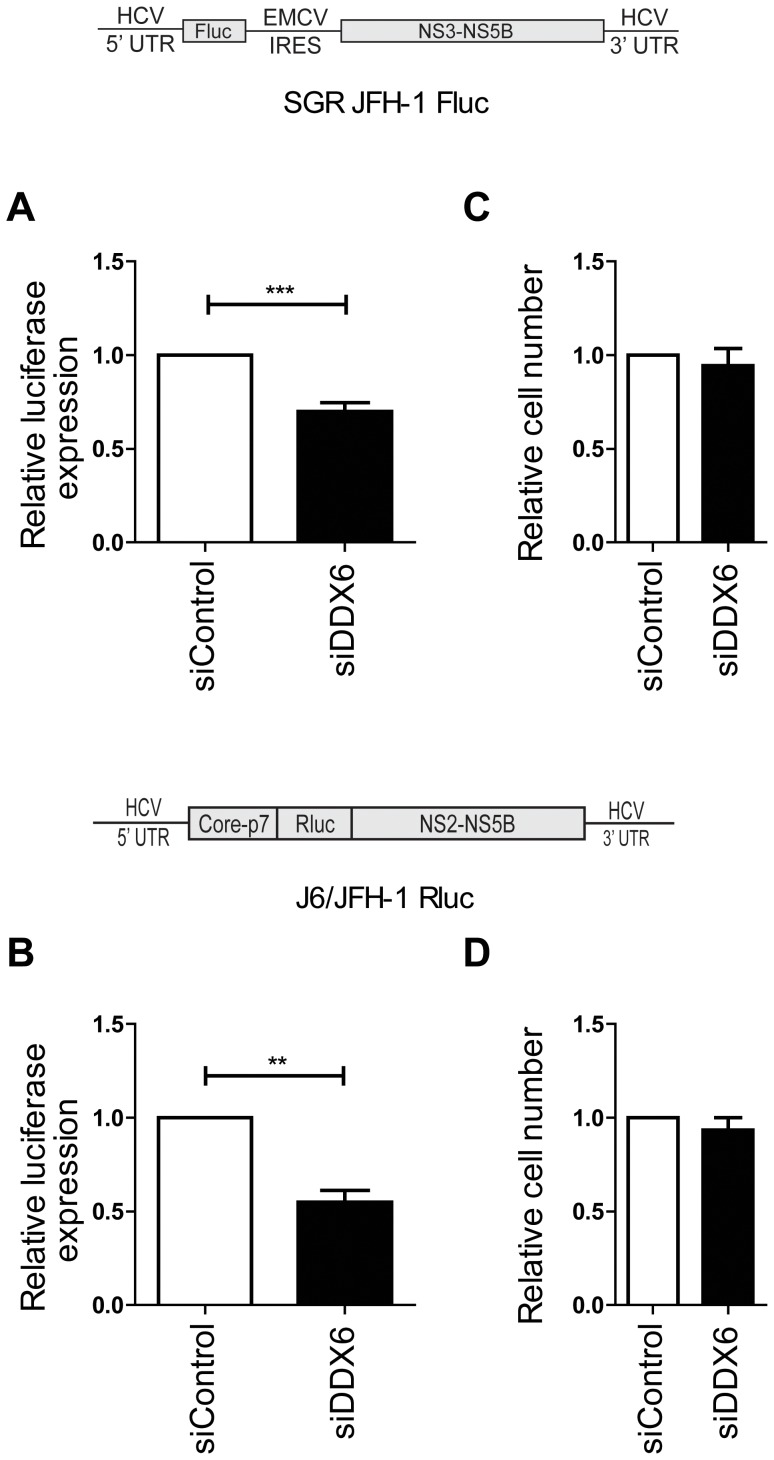
DDX6 depletion attenuates sub-genomic and full-length HCV replication. Relative luciferase expression levels in cells electroporated with (A) sub-genomic JFH-1 Fluc replicon RNA (SGR JFH-1 FLuc), or (B) full length J6/JFH-1 Rluc RNA. (C) Relative cell numbers from A three days after electroporation. (D) Relative cell numbers from B three days after electroporation.

Three days following DDX6 knockdown, replication of both bi-cistronic JFH-1 replicon RNA (SGR JFH-1 FLuc) and full-length J6/JFH-1 RLuc HCV RNA was evaluated. Both RNAs contain luciferase reporter genes so that HCV replication could be evaluated based on luciferase expression. At 3 days post-electroporation luciferase expression from sub-genomic and full-length HCV replicons was decreased by 30% and 45% respectively in DDX6 silenced cells (Fig. 2A and B). Reduced HCV replication was not attributed to a defect in cell proliferation, since WST-1 assays indicated that DDX6 silencing did not significantly affect cell numbers present at the time of harvest (Fig. 2C and D), and DDX6 knockdown was confirmed by western blot (data not shown). These results are similar to, and confirm, those reported by Scheller *et al.*, Jangra *et al.*, and Pager *et al.* [22], [23], [24].

### DDX6 knockdown suppresses HCV translation

**Figure 3:**
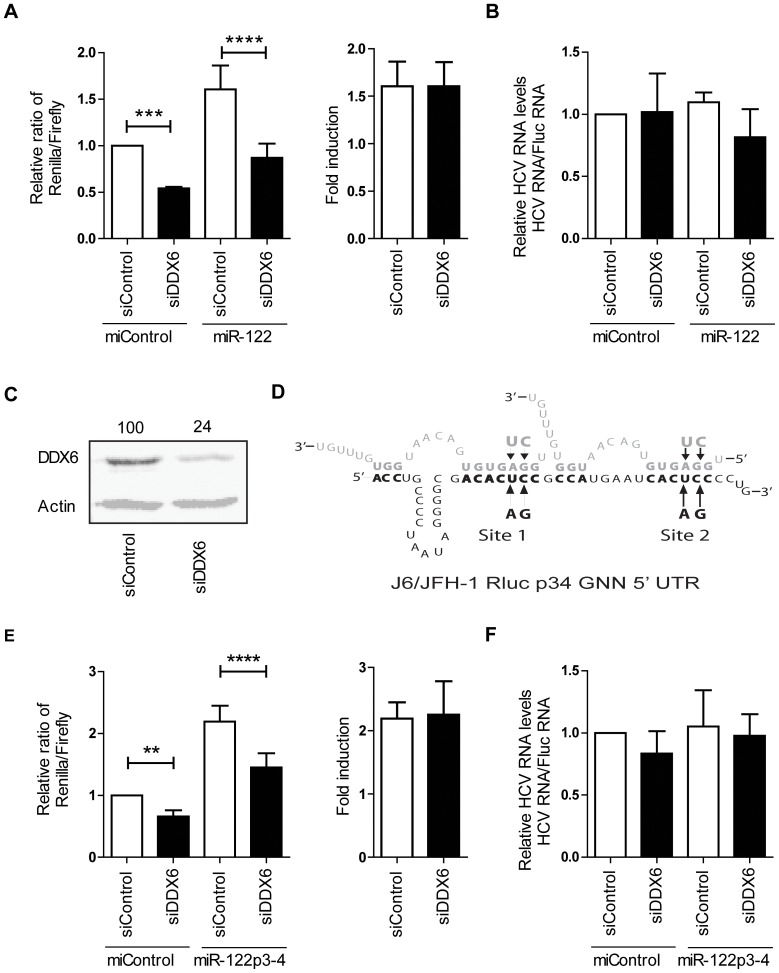
siDDX6 depletion decreases HCV translation, but does not affect miR-122 stimulation of HCV translation. (A) Relative Rluc:Fluc expression in Huh7.5 cells co-electroporated with full-length, replication defective (J6/JFH-1 Rluc GNN) HCV RNA containing a Rluc reporter, a capped Fluc mRNA, and the indicated siRNA and miRNA. The graph on the right shows the relative fold translation stimulation by miR-122. (B) Relative RNA ratios of J6/JFH-1 Rluc GNN to capped firefly mRNA measured by qRT-PCR. (C) Western blot analysis show that siDDX6 depletes cells of DDX6 protein compared to siControl. (D) A schematic drawing of the 5′ UTR of J6/JFH-1 Rluc GNN RNA showing wild-type and mutant miR-122 binding sites (J6/JFH-1 Rluc GNN p34) and annealing pattern with the corresponding miRNA miR-122, or miR-122p34. (E) Relative Rluc:Fluc expression from J6/JFH-1 Rluc GNN p34 RNA co-transfected with capped Fluc mRNA, and the indicated siRNA and miRNA. The graph on the right shows the relative fold translation stimulation by miR-122p34. (F) Relative RNA ratios of J6/JFH-1 Rluc GNN p34 to capped firefly mRNA measured by qRT-PCR. Data in (A) represents the average of 5 independent experiments and the data in (C) represents the average of 8 independent experiments. Significance was determined by performing a one-way ANOVA with Bonferonni's Multiple Comparison Test.

The effect of DDX6 on HCV translation was examined by co-electroporating DDX6-depleted and control cells with non-replicating full-length HCV RNA carrying a Renilla luciferase reporter gene (J6/JFH-1 Rluc GNN) and capped Firefly luciferase mRNA (FLuc). Relative HCV translation levels were determined by calculating the ratio of Renilla luciferase *vs*. Firefly luciferase expression. Knockdown of DDX6 reduced HCV translation of full-length J6/JFH-1 Rluc GNN by 46% compared to cells treated with control siRNA (Fig. 3A, miControl). The phenotype attributed to DDX6 silencing is consistent with that previously reported by *Scheller et al*. [24]. However, we saw inconsistent data regarding whether DDX6 knockdown affected HCV translation. Results obtained using the identical method in an earlier passage of Huh7.5 cells indicated that DDX6 silencing did not affect HCV translation (Fig. S1). The supplementary data are consistent with those reported by Jangra *et al.* [22]. Quantitative RT-PCR quantification of HCV and control Fluc RNA support that the influence of DDX6 and miR-122 on HCV were on translation rather than RNA stabilization (Fig. 3B and Fig. S1B). A western blot confirmed efficient DDX6 knockdown in the cells treated with siDDX6 (Fig. 3C, Fig. S1C).

### DDX6 knockdown does not affect the efficiency of miR-122 stimulation of HCV translation

Since we hypothesized that DDX6 was involved in the activity of miR-122, we expected DDX6 knockdown to attenuate the ability of miR-122 to stimulate HCV translation. To test this we analyzed HCV translation stimulation by miR-122 following knockdown of DDX6. Co-electroporation of miR-122 with J6/JFH-1 Rluc GNN stimulated HCV translation 1.6 fold, and siRNA depletion of DDX6 did not affect the efficiency by which miR-122 stimulated translation (Figure. 3A, miR-122). Thus, DDX6 silencing had no affect on the ability of miR-122 to stimulate HCV translation. To further confirm our observations, we assayed the effect of DDX6 knockdown on HCV translation stimulation by an exogenously provided synthetic miR-122 (miR-122p34). In these experiments, cells were electroporated with J6/JFH-1 Rluc p34 GNN, which contains two point mutations in the miR-122 binding sites, and therefore can no longer bind endogenous miR-122, but can associate with a synthetic miR-122 containing compensatory mutations (Fig. 3D). Knockdown of DDX6 attenuated translation of J6/JFH-1 Rluc p34 GNN by 34%, indicating that miR-122 binding to the HCV genome is not required for the influence of DDX6 on HCV translation (Fig. 3E, miControl). In addition, miR-122p34 stimulated HCV translation by approximately 2 fold in both DDX6 depleted and control cells (Fig. 3E, and S1D, miR-122p34). Quantitative RT-PCR data suggest that the observed effects on translation of J6/JFH-1 Rluc p34 GNN were the result of a decrease in translation and not a decrease in genome stability (Fig. 3F and S1E). From these results we confirmed that the effects of DDX6 and miR-122 on HCV translation are functioning independently.

### DDX6 knockdown does not affect miR-122 augmentation of HCV replication

**Figure 4:**
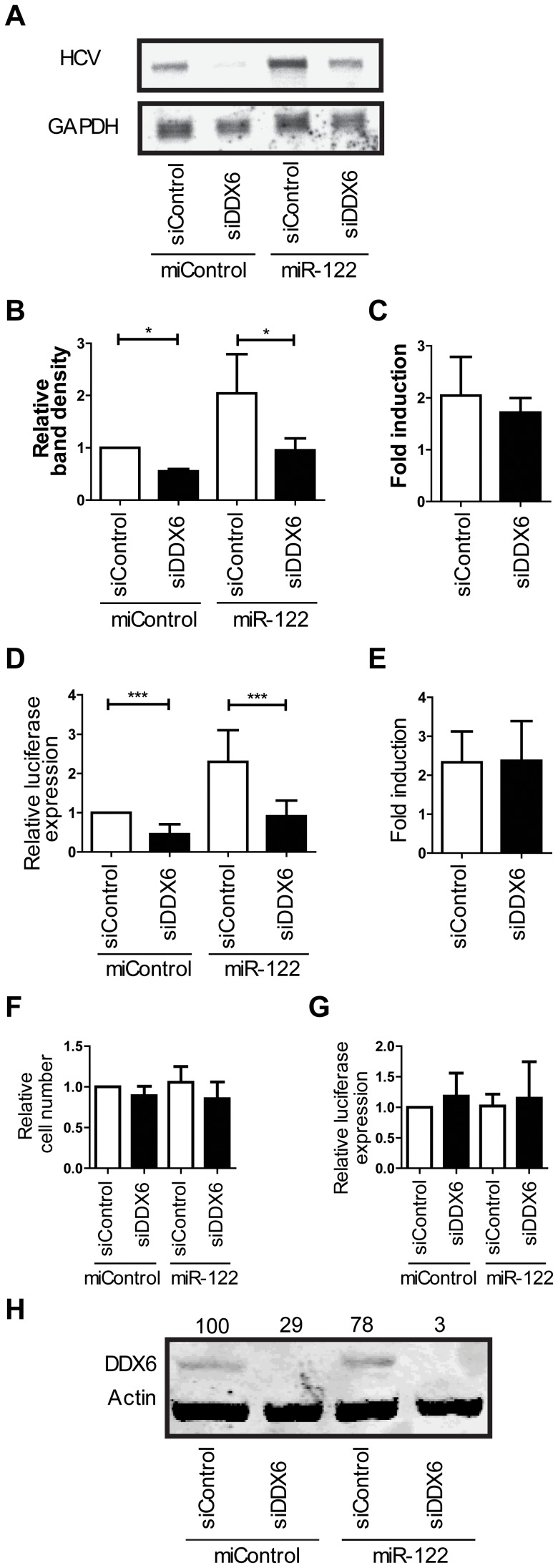
Augmentation of HCV replication by miR-122 is not dependent on DDX6. (A) Representative northern blot analysis of HCV and GAPDH RNA from cells 3 days after electroporation with J6/JFH-1 Rluc RNA and the indicated siRNA and miRNA. (B) Average band intensity measured from 3 independent northern blots as shown in part A, and (C) the corresponding fold induction of RNA accumulation by miR-122. (D) Relative luciferase expression 3 days post-electroporation in the same experiments as shown in parts A, B and C, and (E) the fold induction of luciferase expression by miR-122. (F) Relative cell numbers and (G) Fluc expression from a co-electroporated capped mRNA at 2 hour post electroporation. (H) Western blot analysis of DDX6 protein expression. Data in (D-G) represents the average of 8 independent experiments. A one-way ANOVA with Bonferonni's Multiple Comparison Test was performed on F and G to show they where not significantly different.

Supplementing Huh7.5 cells with miR-122 increases HCV RNA accumulation in infected cells [25], [28], [30], [41]. Since we hypothesize that the role of DDX6 in HCV replication is linked to the activity of miR-122, we expect that the ability of miR-122 to augment HCV replication will be attenuated by DDX6 knockdown. To assess this question we examined the efficiency of miR-122-mediated augmentation of HCV replication with and without DDX6 knockdown by assessing replication-competent HCV RNA accumulation by northern blot and replication by luciferase reporter expression. Northern blot analysis (Fig. 4A and B) confirmed that DDX6 silencing reduced RNA accumulation by 45% in miControl cells, and by 50% in miR-122-treated cells, further supporting the role of DDX6 in enhancing HCV RNA accumulation. When the cells were supplemented with miR-122, J6/JFH-1 RNA levels increased (Fig. 4A and B), confirming that miR-122 supplementation augments HCV accumulation. Importantly, the ability of miR-122 to augment HCV replication was not affected by DDX6 knockdown and miR-122 supplementation increased J6/JFH-1 RNA abundance by about 2.0-fold in siControl and siDDX6-treated cells (Fig. 4C). These observations were confirmed by analysis of luciferase expression where miR-122 supplementation caused a 2.3 fold increase in luciferase expression in both control and DDX6 knockdown cells (Fig. 4D, E). Knockdown of DDX6 did not affect cell growth (Fig. 4F), nor modify translation of a co-electroporated capped Fluc mRNA significantly (Fig. 4G), and DDX6 knockdown was confirmed by western blot analysis (Fig. 4H). These results indicate that DDX6 is not required for miR-122 augmentation of HCV replication.

### DDX6 silencing impedes both miR-122-independent and miR-122-dependent HCV replication in Huh7.5 cells

**Figure 5:**
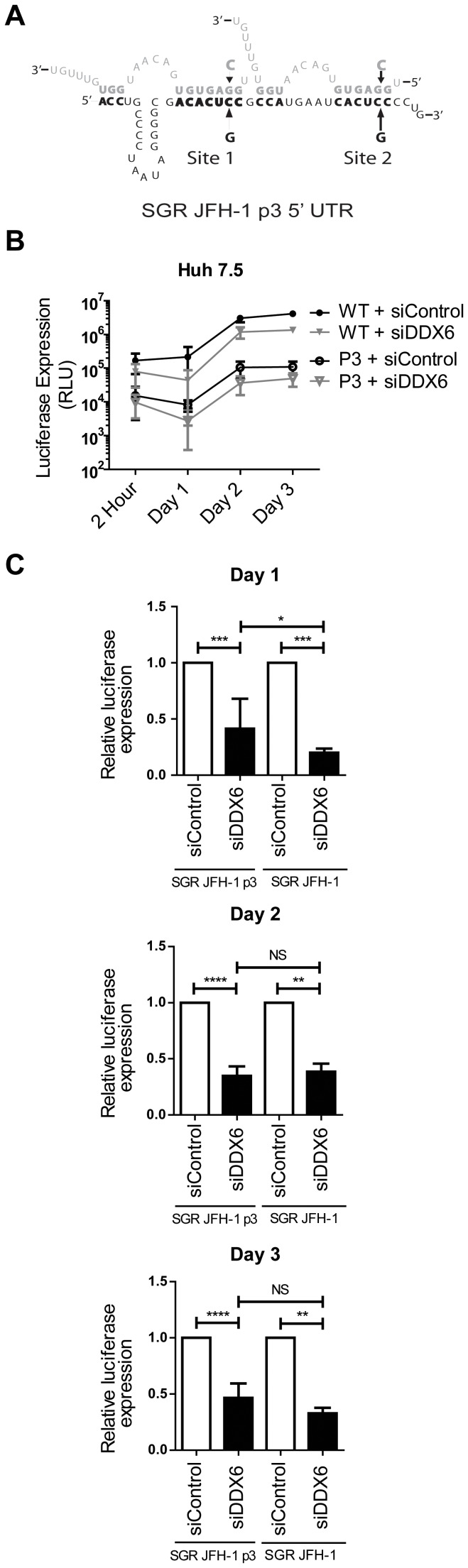
Both miR-122-dependent and miR-122-independent HCV SGR RNA replication in Huh7.5 cells is attenuated by depletion of DDX6. (A) A schematic drawing of the 5′ UTR of the JFH-1 sub-genomic replicon (SGR JFH-1) with a single point mutation in both miR-122 binding sites (SGR JFH-1 p3) that abolishes endogenous miR-122 binding, and the corresponding miRNA containing compensatory mutations to reinstate binding (miR-122p3). (B) Time course of luciferase expression in Huh7.5 cells electroporated with wild type SGR JFH-1 (miR-122-dependent replication) and SGR JFH-1 p3 (miR-122-independent replication) treated with either siDDX6 or control siRNA. (C) Luciferase expression, relative to siControl, on day 1, 2, and 3 post-electroporation for the samples shown in panel B.

We had established that DDX6 is not required for the effects of miR-122 on HCV replication and next wished to determine if the opposite was also true; that miR-122 is not required for the influence of DDX6 on HCV replication. Our lab has recently reported that sub-genomic HCV JFH-1 replicon RNA (SGR-JFH-1) was capable of replicating in Hep3B and Huh7.5 cells without the influence of miR-122 [37]. miR-122-independent HCV replication was confirmed by using replicon RNAs having a single point mutation in both miR-122 binding sites (SGR JFH-1 p3) that renders the replicon unable to bind endogenous miR-122 (Fig. 5A). This system provides a means to screen for genes having a role in the activity of miR-122, since their knockdown should affect miR-122-dependent HCV replication but not miR-122-independent HCV replication. In proof-of-principle experiments, depletion of Ago2, a protein having a role in the activity of miR-122, affected miR-122-dependent but not miR-122-independent HCV replication [37]. We used this system to test whether the effects of DDX6 depletion on HCV replication were dependent on or independent from miR-122 activity. Data indicated that DDX6 functions independently from miR-122 since DDX6 knockdown reduced SGR JFH-1 p3 (miR-122-independent) replication at all three time points analysed (Fig. 5B), and relative to control knockdown cells was 59%, 65%, and 54% lower at days 1, 2, and 3 respectively (Fig. 5C). DDX6 knockdown reduced replication of SGR JFH-1 wt (miR-122-dependent) by 80%, 61%, and 67% on days 1, 2, and 3 respectively (Fig. 5C). The relative decrease of miR-122-independent *vs*. miR-122-dependent HCV replication induced by DDX6 knockdown was not statistically significantly different on days 2 and 3 (Fig. 5C). This data indicates that the effect of DDX6 on HCV replication is not dependent on the activity of miR-122.

### DDX6 silencing impedes both miR-122-independent and miR-122-dependent HCV replication in Hep3B cells

**Figure 6:**
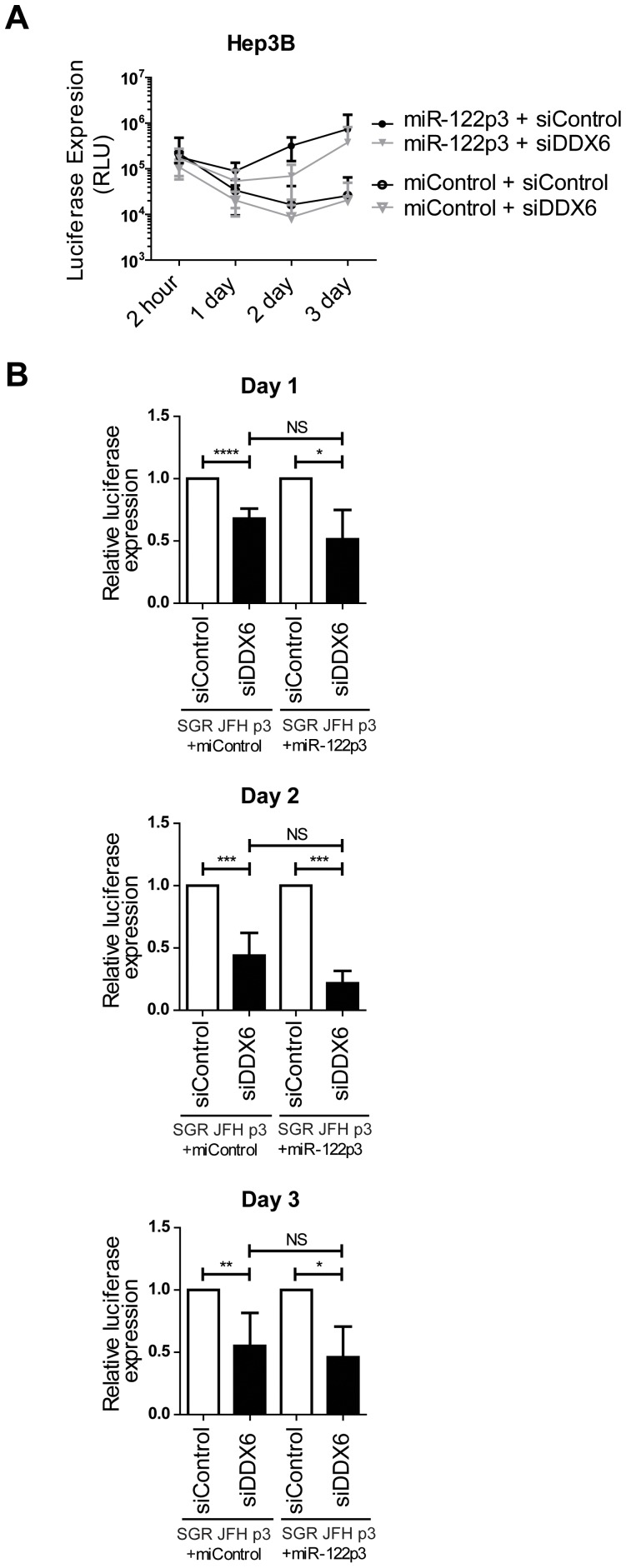
Both miR-122-dependent and miR-122-independent HCV SGR p3 RNA replication in Hep3B cells is attenuated by depletion of DDX6. (A) Time course analysis of luciferase expression from SGR JFH-1 p3 RNA in Hep3Bs cells co-electroporated with either miR-122p3 (miR-122-dependent) or miControl (miR-122-independent) and the indicated siRNAs. (B) Luciferase expression from SGR JFH-1 p3 RNA, relative to siControl, in the presence or absence of miR-122 p3 at days 1, 2 and 3.

We also confirmed the phenotype of DDX6 knockdown on miR-122-independent and miR-122-dependent HCV replication in Hep3B cells. DDX6 knockdown decreased miR-122-independent replication in Hep3B (SGR JFH-1 p3+ miControl) by 32%, 56%, and 45% on days 1, 2, and 3 respectively (Fig. 6 A, B) and was not significantly different from the decrease in replication observed in cells supporting miR-122-dependent HCV replication (SGR JFH-1 p3+ miR-122p3), which were 49%, 78%, and 54%. This data confirms that DDX6 depletion affects HCV replication independent of miR-122, and demonstrates that the influence of DDX6 on HCV replication is not specific to Huh7.5 cells.

### miRNA translation suppression is slightly attenuated by DDX6 knockdown

**Figure 7:**
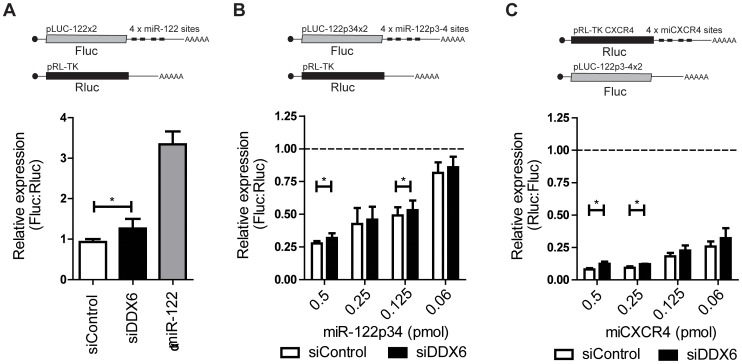
miRNA translation suppression by endogenous and exogenous miRNA is alleviated by DDX6 silencing. (A) Schematic diagram of the mRNAs expressed from the co-transfected reporter plasmids used in this miRNA suppression assay. Expressed mRNAs carry the Fluc sequence with 4 wild-type miR-122 binding sites in the 3′ UTR, or a control Rluc sequence. Relative Fluc:Rluc expression from the reporters was assessed in control, DDX6-depleted, and miR-122 antagonist-treated Huh7.5 cells. (B) Schematic diagram of the mRNAs expressed from the co-transfected reporter plasmids used in this miRNA suppression assay. Expressed mRNAs carry the Fluc reporter gene and 4 mutant miR-122p34 binding sites in the 3′ UTR, or a control Rluc gene. Relative Fluc:Rluc expression from the reporters was assessed in control or DDX6-depleted cells, co-transfected with the indicated amounts of miR-122p34. (C) Schematic diagram of the mRNAs expressed from the co-transfected reporter plasmids used in this miRNA suppression assay. Expressed mRNAs contain an Rluc gene with 4 miCXCR4 miRNA binding sites in the 3′ UTR or a control Fluc gene. Relative Rluc:Fluc expression from the reporters was assessed in control or DDX6-depleted cells that were co-transfected with the indicated amounts of miCXCR4. Data in A represents the average of 6 experiments and B represents the average of 4 experiments.

Others have reported that siRNA knockdown of DDX6 leads to a decrease in the ability of miRNA to silence their targeted genes [14]. To confirm an effect of DDX6 on miRNA suppression activity in Huh7.5 cells, we tested silencing activity of endogenous miR-122, exogenous miR-122p34, and exogenous miCXCR4 in a plasmid-based miRNA suppression assay, with and without knockdown of DDX6 (Fig 7). For the assays, cells were co-transfected with plasmids encoding luciferase (Fluc or Rluc) bearing miR-122, miR-122p34 or miCXCR4 miRNA target sites in the 3′UTR (Fig. 7A, B, and C; top row) and a control plasmid expressing the opposite luciferase, either Fluc or Rluc, to control for transfection efficiency (Fig. 7A, B, and C; top row). Knockdown of DDX6 increased reporter expression by 35% compared to control cells (Fig. 7A) indicating that DDX6 silencing attenuated suppression by endogenous miR-122. In a positive control, co-transfection of a miR-122 antagonist increased luciferase expression by 350% and confirmed that endogenous miR-122 suppressed translation of the reporters (Fig. 7A, αmiR-122). To confirm that the effects of DDX6 knockdown on miRNA suppression was not due to a decrease in miR-122 biogenesis, we also tested DDX6 knockdown using a system in which gene suppression was induced by serial dilutions of an exogenously provided synthetic miR-122p34 (Fig. 7B). In this assay, increased amounts of transfected miR-122p34 caused greater suppression of luciferase expression, and DDX6 knockdown attenuated suppression by miR-122p34 by 11% and 8% when 0.5 and 0.125 pmol of miR-122p34 was used, but not significantly with other dilutions (0.25 and 0.06 pmol) miR-122p34 (Fig. 7B). Although DDX6 knockdown showed statistically significant alleviation of miR-122 silencing, the effect was modest and the physiological relevance questionable. To confirm that our observations were not specific to suppression by miR-122, we also analysed the effects of DDX6 knockdown on miRNA suppression by another miRNA, miCXCR4, using a reporter plasmid containing miCXCR4 binding sites. This assay is identical to one used previously to identify a link between miRNA suppression activity and DDX6 [14]. We observed statistically significant alleviation of miRNA suppression by miCXCR4 following DDX6 knockdown (Fig. 7C), but the effects were relatively small, and not as robust as those previously reported [14], which suggests that DDX6 is not essential for miRNA suppression activity in Huh7.5 cells.

## Discussion

It has been known for several years that both DDX6 and miR-122 support HCV replication. miR-122 functions to support HCV replication by using at least some of the miRNA pathway proteins, with Ago2 having a key role [25], [32], [41]. Since DDX6 interacts with Ago proteins, and both are abundant in P-bodies, we hypothesized that the functions of DDX6 and miR-122 would be linked. However, in spite of the close association of Ago2 and DDX6, and their shared implication in the mechanism of miRNA silencing, extensive evidence indicates that the primary mechanisms by which DDX6 regulates HCV translation and replication is not related to the role of miR-122. Specifically, DDX6 is not required for the effects of miR-122 on HCV replication, and miR-122 association with the HCV genome is not required for the effects of DDX6 on HCV translation and replication. Our data support those of Jangra *et al*, who reported that DDX6 was not required for miR-122 augmentation of HCV replication [22], and we further confirm that DDX6 is also dispensable for miR-122 stimulation of HCV translation. In addition, we confirm that miR-122 is not required for DDX6 to influence HCV replication by showing that DDX6 knockdown still attenuates HCV replication in our miR-122-independent HCV replication assays. However, because there is a general trend showing that DDX6 knockdown affects miR-122-dependent replication slightly more strongly than miR-122-independent replication, and at one time point the difference was statistically significant (Fig. C, 1 day), we cannot exclude the possibility that a second function, requiring both miR-122 and DDX6, has a minor role in supporting HCV replication.

Our studies support the findings of Scheller *et al.*, that DDX6 regulates HCV translation [24], however, in experiments performed in a different passage of Huh7.5 cells, DDX6 silencing had no effect on HCV translation, and our data were similar to those reported by Jangra *et al.* [22]. We cannot explain why HCV translation in different passages of Huh7.5 cells has different requirements for DDX6, but variation in the phenotype and the efficiency by which Huh7-derived cells support HCV replication during Huh7.5 cell passage has been well documented [43], [44]. Regardless of whether DDX6 knockdown does or does not affect HCV translation, in both cases miR-122 was equally capable of stimulating HCV translation in control and DDX6 depleted cells, and thus all of our translation data supports the conclusion that DDX6 is dispensable for miR-122 stimulation of HCV translation.

In addition, our data does not indicate that DDX6 plays a major role in the mechanism of miRNA suppression, which was previously observed in HeLa cells [14]. DDX6 knockdown resulted in a statistically significant attenuation of miRNA gene silencing activity; however, the physiological significance of a role for DDX6 in miRNA suppression activity in Huh7.5 cells is questionable since DDX6 only attenuated suppression by a small amount. Nonetheless, we cannot rule out the possibility that other proteins present in Huh7.5 cells have redundant functions in mediating miRNA suppression, which may explain the discrepancy between our results and those reported in HeLa cells.

The mechanisms of action of DDX6 and miR-122 in supporting HCV replication remain unclear. miR-122 is believed to modulate the efficiency of HCV RNA accumulation by stabilizing genomic RNA [33], [34]. This is likely mediated by miR-122 masking and thus protecting the uncapped 5′ end of the viral genome from degradation by Xrn-1, another P-body protein, but a direct role for miR-122 in the process of HCV replication has also been suggested [33], [34]. In addition, the influence of Xrn-1 knockdown on HCV replication is variable. In some cases, siRNA knockdown of Xrn-1 has been reported to increase HCV replication [33] and in other cases it was reported to have no effect or to decrease HCV replication [21], [23], [24]. These data suggest that perhaps P-body proteins have multiple functions in up-regulating and down-regulating HCV replication.

HCV infections alter P-body structure and recruit P-body proteins such as DDX6, Lsm-1, Pat-1, Xrn1, and Ago2, to lipid droplets and sites of HCV replication [20], [21], [23], [45]. Gene knockdown studies indicate roles for several of these proteins in supporting HCV RNA accumulation and recent evidence indicates that re-localization of P-body proteins during virus infections is not unique to HCV. The yeast DDX6 homolog, Dhh1 is required to recruit Brome mosaic virus genomic RNA to sites of replication in a yeast model replication system [46]. In addition, other members of the *Flaviviridae* family including Dengue and West Nile Virus also disrupt P-body structure and recruit P-body proteins to replication sites, to positively regulate virus replication [45], [47]. Thus, DDX6 and other P-body proteins may have a common role in supporting virus life cycles [45], [47]. That other Flaviviruses utilize DDX6 to support their life cycles, but are not modulated by miR-122 (or by other miRNAs that we know of), also supports the notion that the role of DDX6 (and perhaps other P-body proteins) is not linked to the activity of miR-122. However, we cannot omit the possibility that re-localization of P-body proteins may support Flavivirus replication by using mechanisms that overlap those of miR-122.

Biochemical characterization of DDX6 reveals a possible function in the life cycle of viruses. DDX6 binds to mRNA without sequence specificity, and relaxes its secondary structure [10]. This activity requires ATP binding but not ATP hydrolysis in a way resembling RNA chaperones that stabilize RNA [10]. In a model proposed by Ernoult-Lange *et al.*, DDX6 binds to an mRNA, first as part of a translation repression complex, and then as individual proteins that coat translation-stalled mRNA, and unfolds it in preparation for degradation in P-bodies [10]. DDX6 association with Dengue virus stem-loops, the DB1 and DB2 structures, in the 3′ UTR, is required for efficient virus replication [47]. We speculate that DDX6 could associate with and unfold virus genomes in preparation for initiation of genome replication, however thus far DDX6 has only been reported to associate with HCV genomes through association with the HCV core protein.

DDX6 has also been implicated in the efficiency of HCV virion release and in the assembly of HIV virions [18], [23]. The possible role of DDX6 in HCV virion assembly must be separate from its activity in promoting replication since its knockdown attenuates replication of subgenomic HCV replicons, which do not express core nor assemble particles. However, the association of DDX6 with HCV core protein, and with lipid droplets, could perhaps suggest a role in remodelling viral genome in preparation for virion assembly.

## Supporting Information

Figure S1 **In a subset of experiments we observed that HCV translation was not inhibited by DDX6 knockdown.** (A) Relative luciferase expession of J6/JFH-1 Rluc after electroporation with siDDX6 or siControl. (B) Relative RNA ratios of J6/JFH-1 Rluc GNN to capped firefly mRNA measured by qRT-PCR. (C) Western blot analysis of cell lysates confirming knock down of DDX6. (D) Relative luciferase expression of J6/JFH-1 Rluc m34 in presence and absence of miR-122 p34. The graph on the right shows the relative fold translation stimulation by miR-122p34. (E) Relative RNA ratios of J6/JFH-1 Rluc GNN p34 to capped firefly mRNA measured by qRT-PCR.
(TIF)
