# Supplementary figures and images for: Modulation of Hepatitis C Virus RNA Accumulation and Translation by DDX6 and miR-122 Are Mediated by Separate Mechanisms
Source: PLoS One. 2013 Jun 24;8(6):e67437. doi: 10.1371/journal.pone.0067437 (PMC3691176; doi:10.1371/journal.pone.0067437)

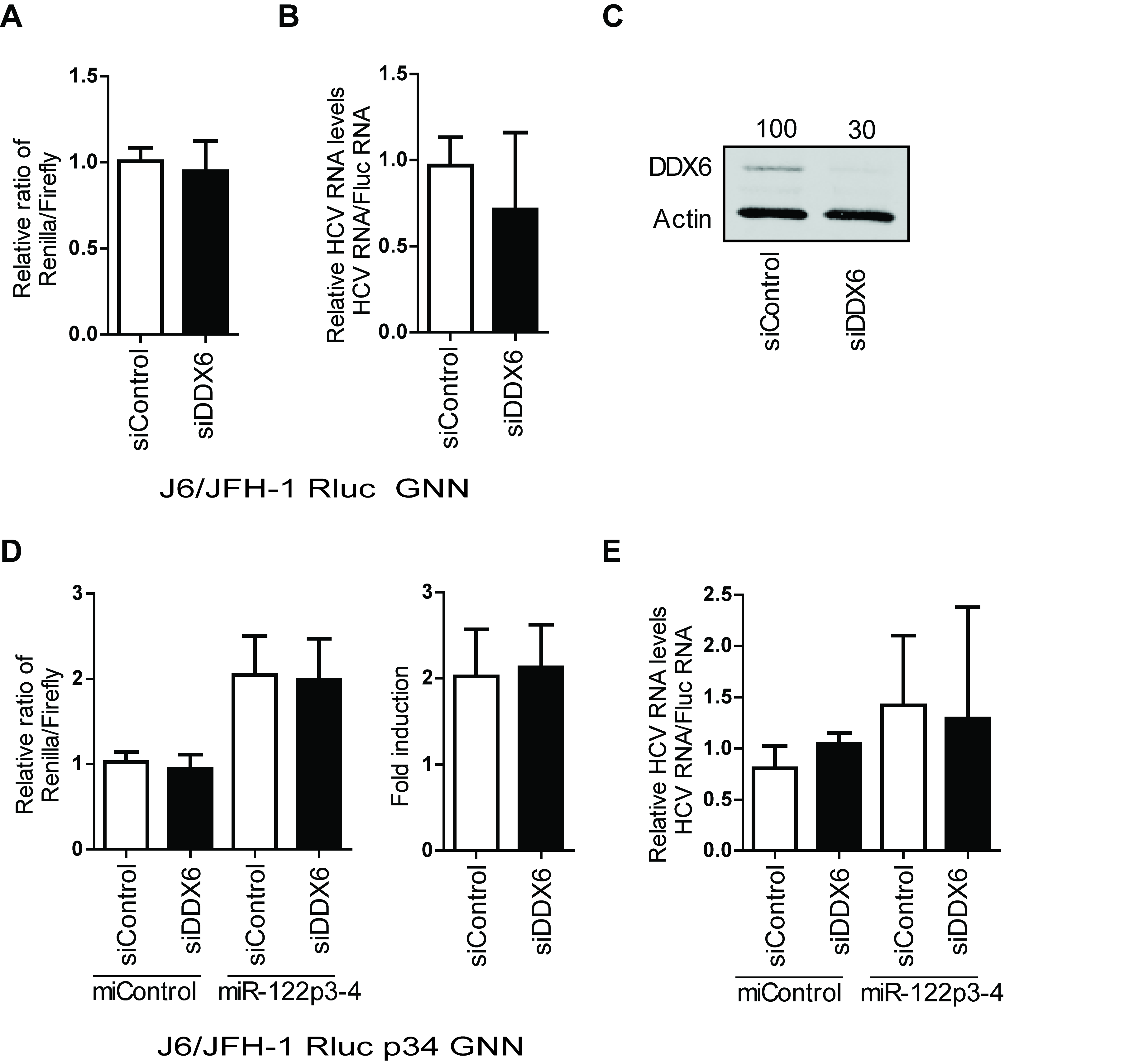

Supplement: Figure S1 — In a subset of experiments we observed that HCV translation was not inhibited by DDX6 knockdown. (A) Relative luciferase expession of J6/JFH-1 Rluc after electroporation with siDDX6 or siControl. (B) Relative RNA ratios of J6/JFH-1 Rluc GNN to capped firefly mRNA measured by qRT-PCR. (C) Western blot analysis of cell lysates confirming knock down of DDX6. (D) Relative luciferase expression of J6/JFH-1 Rluc m34 in presence and absence of miR-122 p34. The graph on the right shows the relative fold translation stimulation by miR-122p34. (E) Relative RNA ratios of J6/JFH-1 Rluc GNN p34 to capped firefly mRNA measured by qRT-PCR. (TIF) [file pone.0067437.s001.tif]
